# Effect of Cognitive Control on Age-Related Positivity Effects in Attentional Processing – Evidence From an Event-Related Brain Potential Study

**DOI:** 10.3389/fpsyg.2021.755635

**Published:** 2021-12-01

**Authors:** Haining Liu, Yanli Liu, Xianling Dong, Haihong Liu, Buxin Han

**Affiliations:** ^1^Department of Psychology, Chengde Medical University, Chengde, China; ^2^Hebei Key Laboratory of Nerve Injury and Repair, Chengde Medical University, Chengde, China; ^3^Department of Biomedical Engineering, Chengde Medical University, Chengde, China; ^4^Centre for Research in Psychology and Human Well Being, Faculty of Social Sciences and Humanities, The National University of Malaysia, Bangi, Malaysia; ^5^Key Laboratory of Mental Health, Institute of Psychology, Chinese Academy of Sciences, Beijing, China; ^6^Department of Psychology, University of Chinese Academy of Sciences, Beijing, China

**Keywords:** cognitive control, go/no-go detection task, age-related positivity effects, ERP, attentional processing

## Abstract

Studies investigating age-related positivity effects during facial emotion processing have yielded contradictory results. The present study aimed to elucidate the mechanisms of cognitive control during attentional processing of emotional faces among older adults. We used go/no-go detection tasks combined with event-related potentials and source localization to examine the effects of response inhibition on age-related positivity effects. Data were obtained from 23 older and 23 younger healthy participants. Behavioral results showed that the discriminability index (*d*') of older adults on fear trials was significantly greater than that of younger adults [*t*(44)=2.37, *p*=0.024, Cohen’s *d*=0.70], whereas an opposite pattern was found in happy trials [*t*(44)=2.56, *p*=0.014, Cohen’s *d*=0.75]. The electroencephalography results on the amplitude of the N170 at the left electrode positions showed that the fear-neutral face pairs were larger than the happy-neutral ones for the younger adults [*t*(22)=2.32, *p*=0.030, Cohen’s *d*=0.48]; the older group’s right hemisphere presented similar tendency, although the results were not statistically significant [*t*(22)=1.97, *p*=0.061, Cohen’s *d*=0.41]. Further, the brain activity of the two hemispheres in older adults showed asymmetrical decrement. Our study demonstrated that the age-related “positivity effect” was not observed owing to the depletion of available cognitive resources at the early attentional stage. Moreover, bilateral activation of the two hemispheres may be important signals of normal aging.

## Introduction

The term “positivity effect” was first put forward by Charles et al. (2003); it describes the age-related differences in facial emotion processing. Evidence has shown that young adults show a selective preference for negative emotional expressions (e.g., angry) compared to positive ones (LoBue et al., 2014). In contrast, they found a positive dominance (e.g., happy expressions) and negativity suppression among older adults (Carstensen and Deliema, 2018). Thus, evidence shows an age-by-valence shift toward positive facial expression processing, shifting away from negative processing that increases with age.

The age-related differences in attentional preferences can possibly be explained by the theoretical framework of socioemotional selectivity theory (SST; Charles and Urban, 2015). The SST is a motivation-related life span theory that posits age differences in goals and are caused by shrinking future time horizons. Young individuals perceive future time as expansive and prioritize information-focused goals (e.g., concern about knowledge acquisition) to prepare for the vague and remote future. However, older individuals perceive the future as more limited; thus, their emotion-related goals (e.g., enjoying intimacy) become more important than all other pursuits in their lives. Carstensen et al. proposed the SST in 1999. Since then, it has been widely applied in social, emotional, health, and cognitive domains (Hicks et al., 2012; Chang et al., 2015; Uzer and Gülgöz, 2015). Moreover, three theories focused on age differences in cognitive and emotional functions have been derived from the SST – the positivity effect (PE), cognitive control hypothesis (CCH), and strength and vulnerability integration – which are generally referred to as second-generation socioemotional selectivity theories (SGSST; Charles and Hong, 2017).

The CCH states that cognitive effort is necessary for older adults to prioritize emotional goals. According to the SGSST, the emergence of age-related PE is because older adults’ top-down goal-driven views will occupy their cognitive resources. While only older adults with sufficient cognitive resources will show positive gaze preference, those with limited cognitive resources are more likely to screen negative emotions. Several studies support the SGSST in that age-related PE results from regulation strategies that occur at late stages of processing and require full cognitive control. For example, Knight et al. (2007) used eye-tracking to reveal that older adults allocate less visual attention to photographs of negative faces under a full attention condition versus a divided attention condition. In a recent study that used a higher cognitive demand task, a reversed fixation pattern was observed in younger adults. They showed longer fixations for happy faces relative to angry faces; however, no difference was found in older adults owing to a decline in inhibitory processing (Bucher et al., 2019). These behavioral findings suggest that cognitive control is essential for age-related PE in older adults’ information processing. The abovementioned research findings suggest that this effect is due to regulation strategies occurring at late stages of processing and require full cognitive control. Mather (2016) suggested that age-by-valence effects are modulated by the medial prefrontal cortex (PFC), which has been associated with detecting affective conflicts and evaluating emotional significance to reach cognitive control of emotional information.

Furthermore, the dynamic integration theory (DIT), another competing theoretical framework, explains PE from the perspective of age-related cognitive decline and neural degradation. The DIT believes that the relationship between cognition and emotion is inherently dynamic. This theory holds that negative information is more complex than positive information and is more difficult to integrate into the cognitive-affective system. Older adults focus on the positive aspects of the environment rather than its negative aspects; this reflects the principle of simple emotional processing. In other words, faced with declining cognitive functions, older adults compensate by distorting information in a positive direction and then evaluate the world simply and positively, thereby processing positive emotional information automatically (Labouvie-Vief, 2009; Bengtson and Settersten, 2016). These arguments indicate that cognitive control ability plays a crucial role in age-related PE. The distinction between automatic and controlled processing depends on the allocation of attentional resources. Some scholars have proposed that, according to the SST, the emergence of PE relies on controlled (late) attentional processes, whereas the DIT assumes that such an effect involves automatic (early) attentional processes (Gronchi et al., 2018).

Recent evidence suggests that the emergence of only a selective positive bias (Madill and Murray, 2017) depends on cognitive control resources; this bias occurs in the early stage of visual processing (Kennedy et al., 2019). Thus, the cognitive mechanisms and attentional stage underlying PE remain unclear. Evidence is likewise lacking on whether attentional preference toward positive face expressions is effortless or dependent on cognitive control resources and whether the early attentional stage is mainly responsible for the onset of such phenomenon.

Event-related brain potentials (ERPs) with millisecond time-resolution measurement can record the time course of neuronal activity patterns involved in various cognitive processes. ERPs are also especially efficient in capturing the rapid shift of early visual attention. Traditionally, the three visual ERP components during the first 200ms after stimulus presentation are considered preconscious and automatic processing, related to emotional effects (Dehaene and Changeux, 2011). Some researchers believe that age-related impairments in structural encoding for facial expressions (Zhao et al., 2016) arise in part from difficulties in the earliest perceptual stages of visual information processing (Gao et al., 2009; Boutet et al., 2020). Nevertheless, beyond well-known age-related cognitive deficits, which might impact emotion identification, the neural temporal dynamics of processing emotional faces are unclear.

P1, which is a positive wave that peaks at approximately 100ms poststimulus presentation at lateral occipito-parietal sites (Rossion et al., 2003; Rossion and Caharel, 2011), is elicited by face processing. This component has also been shown to be modulated by emotion types (Hammerschmidt et al., 2017; Schindler et al., 2019) in healthy young adults. Recent ERP research has reported age-related PE. For instance, Hilimire et al. (2014) asked young and older adults to perform the checkerboard probe go/no-go task where the faces have angry, sad, happy, and neutral expressions over the time window of P100. Their results also showed younger adults having an enhanced frontocentral neural activity elicited by negative emotional expressions. In contrast, older adults have an enhanced frontocentral neural activity elicited by positive emotional expressions.

The posterior temporal N170 – a negative wave peaking between 130ms and 200ms, which is commonly identified as a valuable face-sensitive electrophysiological index (Hinojosa et al., 2015). This sensitivity is heterogeneous, with enhanced N170 amplitudes elicited by emotional faces compared with neutral ones. For example, Liao et al. (2017) conducted a study where participants performed a facial emotion categorization task and found that, compared with younger counterparts, older adults show an enhanced N170 for negative faces. Mienaltowski et al. (2015) presented participants with facial expressions under four conditions – passively view, passively view but consider emotions, categorize emotions, and categorize sexes. However, the study failed to find the emergence of the age-by-valence PE pattern. They concluded that cognitive functioning in older adults may be related to structural encoding deficits for negative faces. In addition, the typical lateralization effect of the N170 in younger adults, with larger amplitudes located in the right hemisphere than in the left hemisphere, was attenuated in older adults (Bentin et al., 1996; Eimer et al., 2011). Across these studies, conflicting evidence is presented on whether age-related PE emerges during early attention processing. Furthermore, consensus is yet to be reached regarding the P1 and N170 waveforms, related to emotion processing and cognitive aging.

Given the inconsistencies in recent findings, questions arise as to how cognitive control mechanisms for the emergence of PE are measured. Discrepancies in task design (i.e., emotion categorization, and passive view) may be mainly responsible for the contradictory results. A variant of this paradigm combined with the emotion dot-probe task (go/no-go detection task) has been used in ERP research for investigating the effects of response inhibition on attentional processes of emotional information (Pourtois et al., 2004; Santesso et al., 2008). In this paradigm, researchers gather the behavioral measures during go trials and time lock the ERPs to emotional stimuli onset without motor-related artifacts during no-go trials. This paradigm may be promising for disentangling why and when positive emotional preferences shift among older adults in conditions related to response inhibition. Additionally, previous studies used this task to examine hemispheric asymmetries among young, healthy individuals (Pourtois et al., 2004). Few studies have directly tested the age effects of hemispheric asymmetries during the emotional attention process.

This study utilized the go/no-go detection task developed by Pourtois et al. (2004) to examine the effects of response inhibition on visual processing of emotional faces among younger and older adults. ERPs assessed potential age-related differences in neurological responses time-locked to valence faces as a function of the response inhibition imposed by go and no-go secondary tasks. Age-related PE has been associated with cognitive control ability in assessments of visual attention to emotional faces (Mather and Carstensen, 2005; Isaacowitz et al., 2006). We assumed that cognitive control would be essential for age-related PE. Given the evidence that older adults seemingly do not show attentional preferences (Knight et al., 2007) toward positive facial expressions when attentional resources are occupied by secondary tasks, we expected that older participants would show some extent of attentional bias toward threatening cues, similar to their younger counterparts, as manifested by potentiated P1 and face-sensitive N170 amplitudes to fearful faces. Given that younger adults are inclined to focus more on processing negative rather than positive information (Baumeister et al., 2001; Rozin and Royzman, 2001), we hypothesized that regardless of the condition, negative facial expressions would evoke a greater amplitude of visual-evoked potentials relative to positive facial expressions in younger adults. Older adults tended to demonstrate increased activity in related brain areas, such as the anterior cingulate cortex (ACC; Sakaki et al., 2013; Dolcos et al., 2014), during the processing of negative emotional faces.

## Materials and Methods

### Participants

Estimates for the 2 (Between Group)×2 (Within Group) ANOVA were computed using G*Power (version 3; Faul et al., 2009). The number of participants required to achieve a statistical power of 0.80 for effect size (Interaction: Cohen’s *d*=0.482, Cohen’s *f*=0.241, Reed et al., 2014; Age main effect: *η*^2^=0.489, Cohen’s *f*=0.561, Hahn et al., 2006). An alpha level of 0.05 required 36 participants (18 per group) and 22 participants (11 per group) for the interaction and the main effect, respectively. We recruited 26 older adults and 25 undergraduate students to participate in the experiment. Two of them were excluded owing to poor behavioral performance (>50% error trials); three were removed from the study for having excessive artifacts during the electroencephalogram (EEG) signal recording. This resulted in a final sample of 23 older (aged 60–81years; *M*=71.78, *SD*=6.27) and 23 younger participants (aged 19–26years; *M*=22.22, *SD*=1.70), which was adequate for the objective of this study. The educational levels of the two groups were matched. All participants were right-handed, with normal or corrected vision and had no history of neurological or mental illnesses. All scores on the modified Mini-Mental Status Exam of older participants exceeded 27 (*M*=29.21, *SD*=0.95). All participants signed an informed consent form and received some financial compensation. The study was approved by the Institutional Review Board of the Institute of Psychology of the Chinese Academy of Sciences.

### Questionnaires

Before the experiment, participants underwent a series of neuropsychological assessments, including the State-Trait Anxiety Inventory (Shahid et al., 2012), Positive Affect and Negative Affect Scale (Brdar, 2014), Center for Epidemiologic Studies Depression Scale (Teale Sapach et al., 2017), Digit Cancellation Test for Attention (DCT-A), and three subscales of the Wechsler Intelligence Scale for Adults-Fourth Edition (Drozdick et al., 2018), including Knowledge, Arithmetic, and Digit Span. Table 1 presents the demographic characteristics and neuropsychological measurements of younger and older adults. The older group scored significantly lower than the younger group in DCT-A, arithmetic, and digit span tests. This indicates that the older participants exhibited deterioration in continuous attention, digital reasoning, active attention, and working memory span.

**Table 1:** Demographic characteristics and neuropsychological measures (*M*+*SD*).

	Young group (*n* =23)	Older group (*n* =23)	*t/χ* ^2^	*p*	Cohen’s *d*
Age in years	22.22±1.70	71.78±6.27	36.60	<0.001	−10.79
Male/Female (*n*)	10/13	11/12	0.09	0.767	–
Years of education	15.30±1.49	15.04±1.85	0.53	0.601	0.16
STAI-T	53.40±2.65	52.86±7.04	0.35	0.731	0.10
PANAS_P	32.74±6.46	34.96±5.14	−1.29	0.204	−0.38
PANAS_N	17.96±4.49	16.26±3.44	1.44	0.157	0.42
CES-D	30.70±7.53	31.74±6.05	−0.52	0.607	0.16
DCT-A	30.29±4.27	11.47±21.11	4.19	<0.001	1.24
Knowledge	24.44±2.59	24.72±3.12	−0.33	0.740	−0.10
Arithmetic	15.83±1.99	13.09±2.75	3.87	<0.001	1.14
Digit Span	16.70±2.75	13.17±2.04	4.93	<0.001	1.45

### Stimuli

The emotional images were selected from the FACES database (Ebner et al., 2010), a database of facial expressions developed by the Max Planck Institute for Human Development in Germany. We chose happy, fearful, and neutral faces of 16 older (8 men and 8 women) and 16 younger (8 men and 8 women) adults, resulting in a total of 96 emotional faces. Each person had a unique ID code. Each face pair consisted of the same identity portraying an emotional face (happy or fearful) and a neutral face according to the following four conditions: neutral-fearful, fearful-neutral, neutral-happy, and happy-neutral. Each emotional face was presented with equal frequency on the left or right side of the neutral face.

### Experimental Procedures

**Figure 1:**
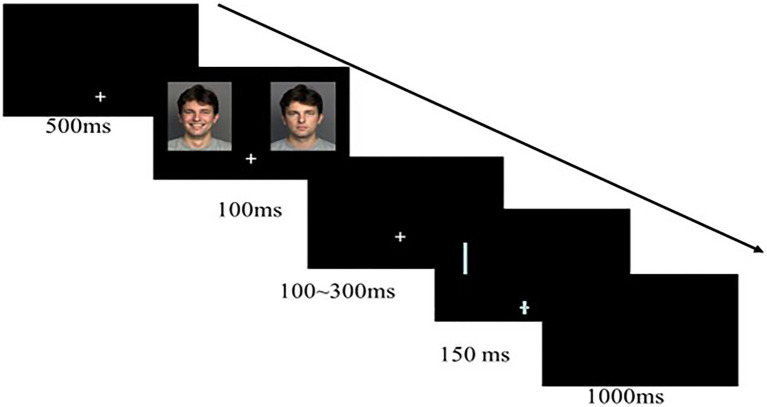
Example trial sequence of the Go/No-go detection task.

The participants performed a go/no-go detection task (Pourtois et al., 2004; Dhar and Pourtois, 2011). An example trial flow is shown in Figure 1. Stimuli were presented on a 17-inch CRT computer screen with a black background. The participants sat before the screen at a viewing distance of approximately 58cm. Each trial started with a fixation cross, measuring 2×2cm with a thickness of 0.1cm, centrally displayed on the lower visual field for 500ms. The face pair (each picture with 8×10 degrees of visual angle) was presented in the upper part of the computer screen, with a distance of 2cm from the horizontal top and bilateral outer edges, and each face was equidistant from the vertical centerline. The face disappeared after 500ms; then, only the fixation cross was presented randomly for 100–300ms, followed by a white rectangular bar (50% horizontal and 50% vertical) probe measuring 7×0.4cm, appearing at either previous location of the two faces (50% left and 50% right). The targets replaced fearful or happy faces, referring to “happy-valid” and “fear-valid,” and replaced neutral faces, referring to “happy-invalid” and “fear-invalid.” Meanwhile, the thickness of either the horizontal or vertical line within the cross was randomly increased by 0.1, 0.2, 0.3, and 0.4cm and then presented simultaneously with the probe target for 150ms. If the orientation of the thicker line at fixation matched the bar in the upper screen, then the participants were asked to press the corresponding key with their right index finger (go trials) in 10% of all cases; otherwise, they were required not to press any button (no-go trials). The inter-trial interval time was 1,000ms. The participants were asked to respond as accurately and quickly as possible.

The entire experiment included practice and formal stages. The practice phase consisted of 16 trials that provided feedback on whether the participants’ responses were correct. A total of 512 trials were conducted in the formal experiment phase, and no feedback was provided. The formal experiment consisted of eight blocks, each containing 64 mixed-emotion trials; that is, a pair of emotional (happy or fearful) faces matched the neutral faces of the same person. Half of the mixed-emotion trials were happy-neutral pairs, and the other half, fearful-neutral pairs. Similarly, half of the emotional faces were presented on the left visual field and the other half on the right. In each block, 8 go trials (2 emotion types×2 probe locations×2 probe orientations) and 56 no-go trials (2 emotion types×2 probe locations×2 probe orientation×7 repetitions) were presented in a pseudo-random manner; the faces with the same ID were not presented in two adjacent trials. Between each block, the participants rested for 1min.

### Electroencephalogram Record and Acquisition

Electroencephalogram data were recorded using a NeuroScan (SynAmps) acquisition system (NeuroScan, Inc., Charlotte, NC, United States) with a 64-channel Ag/AgCl electrode cap in the International 10/20 System positions. The left mastoid served as a reference electrode during online recording and was re-referenced offline to averaged mastoids. The ground electrode was located between FPz and Fz, and impedances were maintained below 5 kΩ. We used a band-pass filter of 0.01–400Hz and a sampling frequency of 1,000Hz to digitize the raw signals. Horizontal eye movements were recorded using a horizontal eye electrooculogram (EOG), monitored by two electrodes placed at the external canthi; eye blinks and vertical eye movements were detected by a vertical EOG, which was recorded from electrodes placed above and below the left orbit.

Analysis of the EEG data was performed using curry 7.0. Corrected trials were low-pass filtered offline using cutoffs of 30Hz. Baseline correction was performed using the 100ms pre-stimulus period. Trial voltages exceeding ±100μV were rejected automatically as artifacts. The remaining artifacts of all trials were visually inspected and rejected per trial. Vertical and horizontal eye movements and eye blinks were corrected using covariance analysis. Epochs were extracted from 100ms before the emotion stimulus onset and lasted for 500ms after the emotion stimulus was presented. Error trials were excluded from the EEG analyses.

To prevent contamination from motor artifacts, we reported and analyzed only visual event-related brain potential (VEP) components evoked by the no-go trials. Two exogenous VEP components time-locked to the face pair onset, including the P1 and N170, were analyzed. After evaluation, P1 was considered the most positive peak in the time window of 80–150ms after face onset at the left and right occipital/posterior sites (channels: left PO5, PO3; right PO4, PO6), which coincides with the study of Luck et al. (1990). Similarly, after evaluation, the N170 was considered the most negative peak in the time window of 130–210ms after face onset at the left and right occipital temporal sites (channels left: PO7, P7, P5, P3; right P4, P6, P8, PO8), which was consistent with a meta-analysis of N170 (Carretie, 2014).

### Source Localization

The locations of the neural generators for the face-specific N170 (130–210ms) were reconstructed using standardized low-resolution brain electromagnetic tomography (sLORETA 2008; Pascual-Marqui, 2002). We conducted the sLORETA on grand-averaged MRI data from the Montreal Neurological Institute (MNI; Talairach and Pa, 1988) based on the three-shell spherical model (Fuchs et al., 2002). We then converted the MNI coordinates into Talairach space (Brett et al., 2002) to identify Brodmann areas and gyri. Next, voxel-wise paired and independent *t* tests for the log-transformed power of the standardized electric current density were calculated to assess the locational differences in cortical activity between the conditions and groups. For each comparison, results corrected for multiple comparisons by 5,000 randomizations were considered to indicate significant brain area activation at the critical threshold of *p*<0.05 (Nichols and Holmes, 2003).

### Statistical Analyses

To quantitatively assess accuracy (i.e., misses in go trials and false alarms in no-go trials), we computed an index of discrimination (*d*') using signal detection theory (Green and Swets, 1966). A higher value of *d*' indicates that the signal is more salient and easier to identify (compared to noise). For *d*' and reaction times (RTs), a repeated measures ANOVA was conducted including emotion types (fear vs. happy) and cue validity (valid vs. invalid) as within factors and age (young group vs. older group) as a between factor. Specially, the RTs with only accurate responses were analyzed.

For ERPs, a repeated measures ANOVA was conducted – including emotion types (fear vs. happy), cue validity (valid vs. invalid), and electrode location (left vs. right) as within factors and age (young group vs. older group) as a between factor – on P1 and N170. All results reported were Greenhouse-Geisser corrected. Significant interactions were further analyzed using *t* tests (two-tailed). Effect sizes are reported as unbiased Cohen’s *d* for *t* tests and as *η*^2^ for ANOVAs.

## Results

### Behavior

For mean RTs on go trials, repeated measures ANOVAs revealed a main effect of age [*F*(1,44)=24.86, *p*<0.001, *η*^2^=0.36]; younger participants completed trials faster than older participants (*M*_young_=325.48±26.17; *M*_old_=509.97±26.17, *p*<0.001).

**Figure 2:**
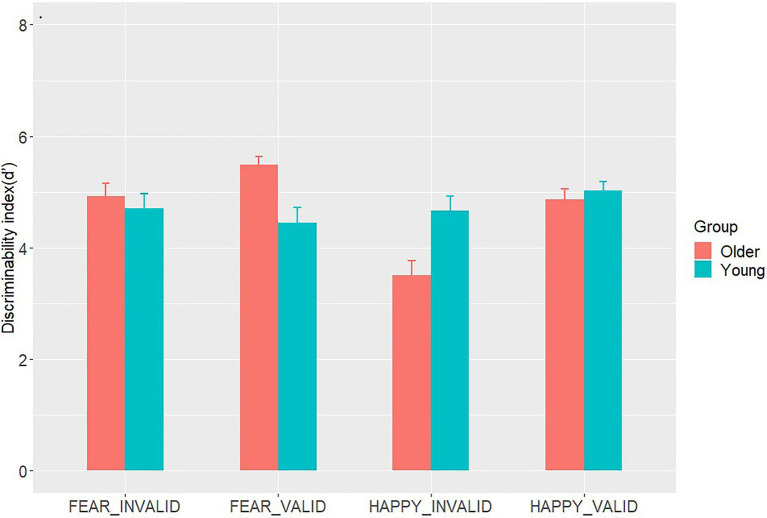
The mean values of *d*' in Go/No-go detection tasks for both age groups. The vertical lines above the bar stand for 1/2 Standard Error.

Repeated measures ANOVAs were conducted on *d*' (Table 2 and Figure 2). As for the main effects of emotion types and cue validity [*F*(1,44)=4.30, *p*=0.044, *η*^2^=0.09; *F*(1,44)=15.88, *p*<0.001, *η*^2^=0.27, respectively], *d*' of the fear trials was significantly better compared with that of the happy trials (*M*_happy_=4.51±0.13; *M*_fear_=4.89±0.13, *p*=0.044); *d*' under the valid condition was also reliably better compared with that under the invalid condition [*M*_valid_=4.96±0.11; *M*_invalid_=4.45±0.12, *p*<0.001]. The ANOVAs revealed significant interactions between age group and emotion type [*F*(1,44)=11.97, *p*=0.001, *η*^2^=0.21], age group and cue validity [*F*(1,44)=12.71, *p*=0.001, *η*^2^=0.22], and emotion type and cue validity interaction [*F*(1,44)=6.28, *p*=0.02, *η*^2^=0.13]. In fear trials, *d*' among older participants (*M*_old_=5.21±0.60) was higher than that among younger participants (*M*_young_=4.58±1.12) [*t*(44)=2.37, *p*=0.024, Cohen’s *d*=0.70]. Meanwhile, an opposite pattern was found in happy trials [*t*(44)=2.56, *p*=0.014, Cohen’s *d*=0.75]. For older adults, *d*' on fear trials (*M*_fear_=5.21±0.60) was higher than that on happy trials (*M*_happy_=4.18±0.89) [*t*(23)=4.57, *p*<0.001, Cohen’s *d*=1.36], whereas *d*' on fear trials (*M*_fear_=4.58±1.12) was not different from that on happy trials for younger adults (*M*_happy_=4.84±0.83) [*t*(23)=0.87, *p*=0.393, Cohen’s *d*=0.26]. In the valid condition, *d*' of the older participants was higher than that of the younger participants [*t*(44)=2.10, *p*=0.041, Cohen’s *d*=0.62]. In the invalid condition, *d*' of the older participants was similar to that of their younger counterparts [*t*(44)=1.99, *p*=0.053, Cohen’s *d*=0.59]. In the fear condition, *d*' in valid trials was similar to that in invalid trials (*M*_valid_=4.94±0.13; *M*_invalid_=4.08±0.20, *p*<0.001). A higher *d*' for valid than invalid trials was specifically observed under the happy condition (*M*_valid_=4.94±0.13; *M*_invalid_=4.08±0.20, *p*<0.001). However, the three-way interaction between age groups, emotion types, and cue validity was not statistically significant [*F*(1,44)=0.10, *p*=0.75, *η*^2^=0.002].

**Table 2:** The mean values of *d*' in Go/no-go tasks for both age groups (ms) (*M*±*SD*).

Emotion	Validity	Young group (*n* =23)	Older group (*n* =23)
Fear	Valid	4.45 ± 1.28	5.49 ± 0.70
	Invalid	4.70 ± 1.24	4.92 ± 1.14
Happy	Valid	5.01 ± 0.82	4.86 ± 0.96
	Invalid	4.66 ± 1.25	3.51 ± 1.18

### P1 Latency

**Figure 3:**
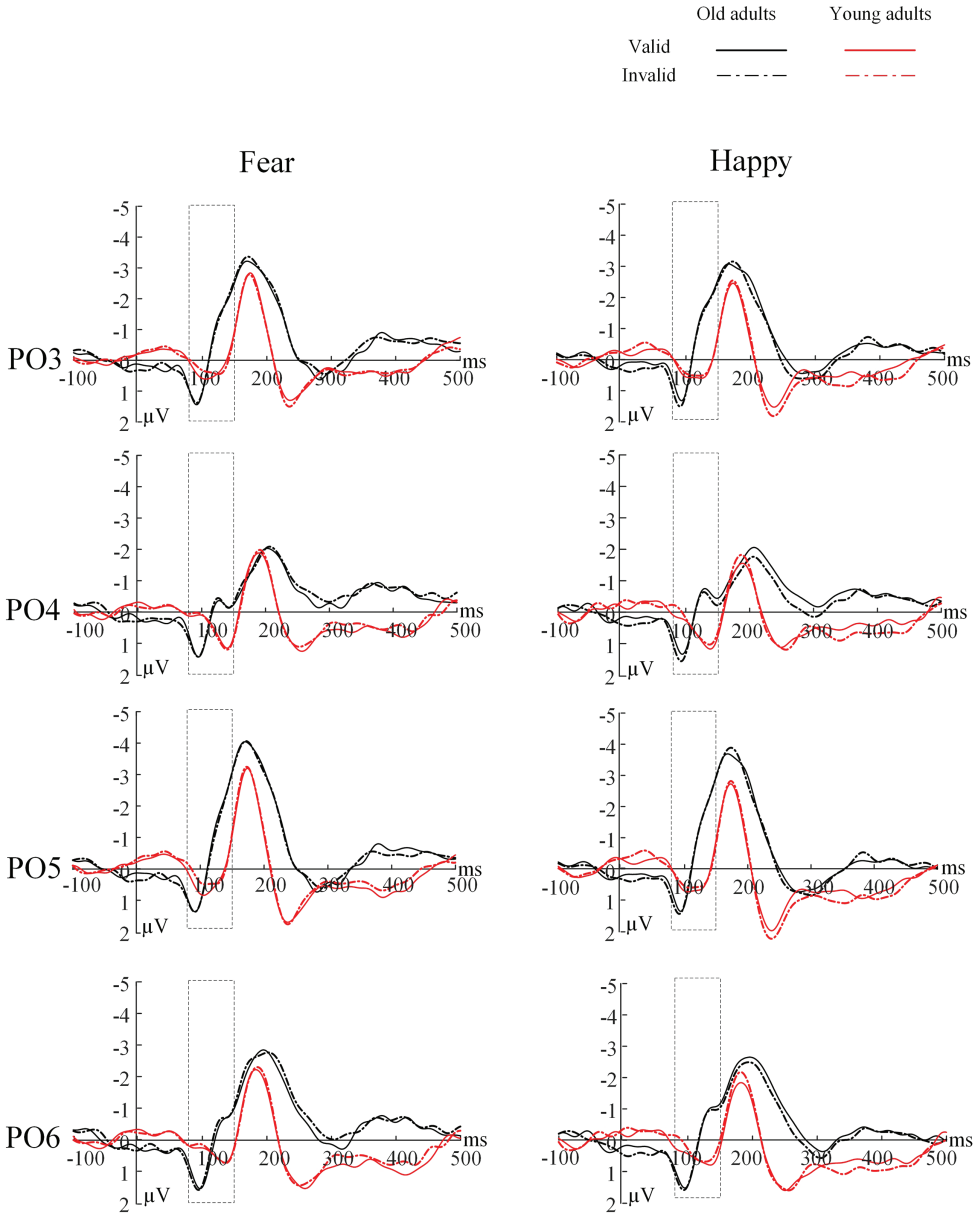
Grand average ERP of the P1 component for each group at channels of PO3/PO4 and PO5/PO6. Solid lines=valid trials, dotted lines=invalid trials, black=older group, red=young group. The rectangular box represents the selected time window.

For the peak latency of the P1 component (Table 3 and Figure 3), a significant main effect of electrode location was found [*F*(1,44)=4.31, *p*=0.044, *η*^2^=0.09], such that the P1 latency was shorter in the left hemisphere than in the right hemisphere (*M*_left_=107.70±2.42; *M*_right_=113.61±2.37, *p*=0.044). No other significant main effects or interactions were found (*Ps*>0.05).

**Table 3:** Mean latency (ms) and mean amplitude (μV) of the P1 (*M*±*SD*).

	Emotion	Validity	Young group (*n* =23)		Older group (*n* =23)	
			Left	Right	Left	Right
Latency	Fear	Valid	114.35 ± 21.95	129.39 ± 18.18	96.83 ± 14.53	104.00 ± 17.89
		Invalid	118.74 ± 20.02	128.20 ± 19.35	99.00 ± 18.89	98.54 ± 15.61
	Happy	Valid	117.98 ± 19.47	124.17 ± 19.70	96.78 ± 14.17	97.91 ± 15.81
		Invalid	122.63 ± 18.80	125.50 ± 19.24	95.28 ± 15.28	101.20 ± 19.90
Amplitude	Fear	Valid	1.90 ± 1.54	1.93 ± 1.65	1.83 ± 1.03	2.06 ± 1.38
		Invalid	1.84 ± 1.70	2.01 ± 1.41	1.80 ± 0.79	2.00 ± 1.13
	Happy	Valid	1.99 ± 1.50	2.12 ± 1.63	1.79 ± 1.15	1.80 ± 1.31
		Invalid	2.07 ± 1.63	1.93 ± 1.54	1.79 ± 0.91	2.02 ± 1.19

### P1 Amplitude

For the peak amplitude of P1 (Table 3 and Figure 3), we found neither significant main effects nor interactions (*Ps*>0.05).

### N170 Latency

**Figure 4:**
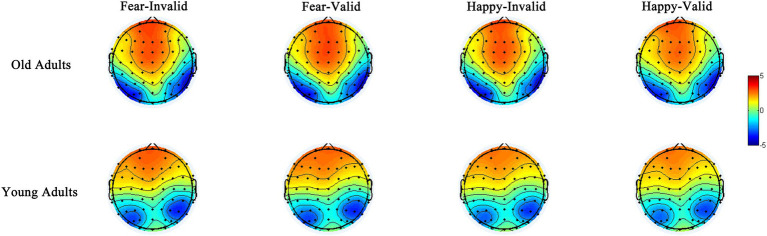
Grand average ERP of the N170 component for each group at channel P3/P4, P5/P6, P7/P8 and PO7/PO8. Solid lines=valid trials, dotted lines=invalid trials, black=older group, red=young group. The rectangular box represents the selected time window.

The ANOVA revealed a significant main effect of electrode location [*F*(1,44)=10.25, *p*=0.003, *η*^2^=0.19], indicating that the peak latency of the N170 was earlier in the left hemisphere than in the right hemisphere (*M*_left_=172.58±2.25; *M*_right_=181.19±2.35, *p*=0.003). We found no other significant main effects or interactions (*Ps*>0.05; Table 4 and Figure 4).

**Table 4:** Mean latency (ms) and mean amplitude (μV) of the N170 (*M*±*SD*).

	Emotion	Validity	Young group (*n* =23)		Older group (*n* =23)	
			Left	Right	Left	Right
Latency	Fear	Valid	173.63 ± 13.50	176.36 ± 16.20	173.66 ± 16.42	184.45 ± 19.61
		Invalid	173.42 ± 14.26	178.23 ± 13.96	170.25 ± 18.91	186.30 ± 19.13
	Happy	Valid	174.04 ± 16.21	114.35 ± 21.95	171.10 ± 17.35	114.35 ± 21.95
		Invalid	173.50 ± 14.21	176.99 ± 14.94	171.02 ± 19.15	183.35 ± 18.86
Amplitude	Fear	Valid	−3.11 ± 2.44	−3.78 ± 1.86	−4.02 ± 2.55	−3.88 ± 2.18
		Invalid	−3.23 ± 2.06	−3.79 ± 1.99	−4.01 ± 2.43	−4.09 ± 2.36
	Happy	Valid	−2.89 ± 2.11	−3.62 ± 2.10	−4.03 ± 2.72	−3.89 ± 2.02
		Invalid	2.07 ± 1.63	1.93 ± 1.54	1.79 ± 0.91	2.02 ± 1.19

### N170 Amplitude

**Figure 5:**
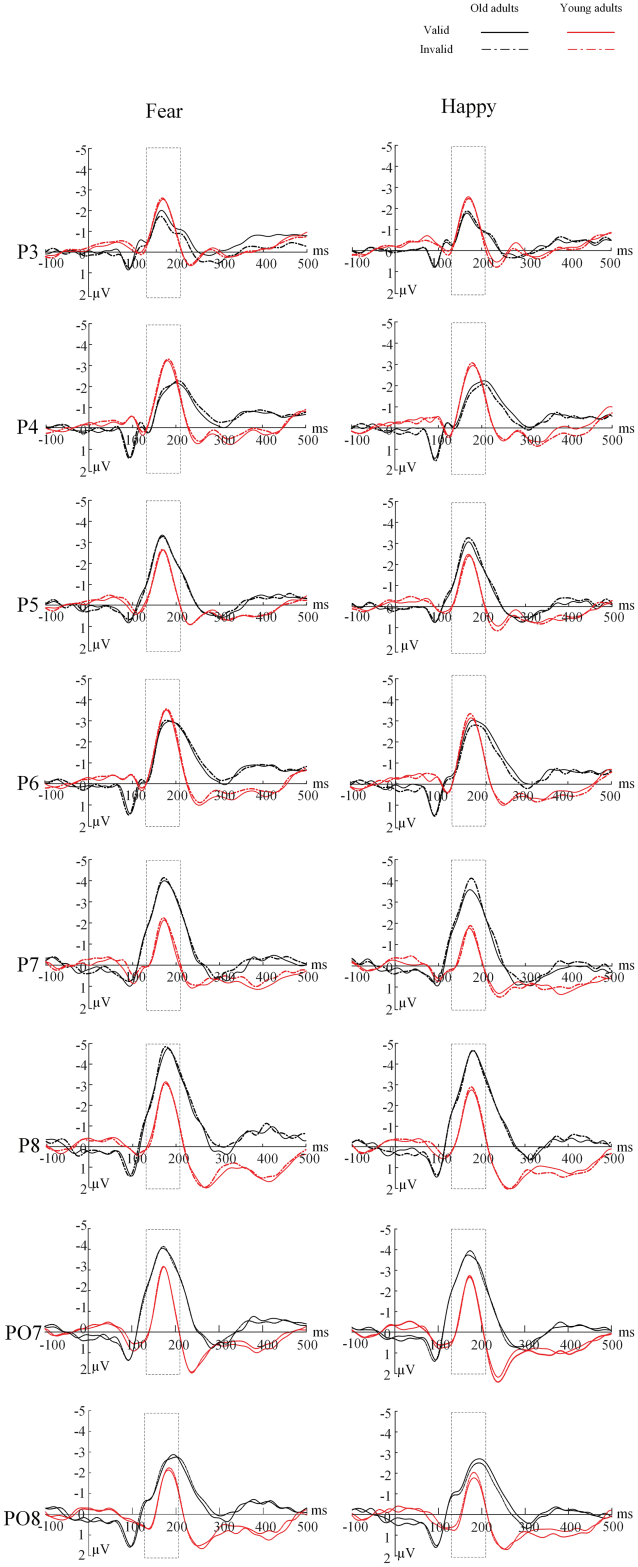
The topographic maps for N170 for both old and young groups for four conditions: Fear – Invalid, Fear – Valid, Happy – Invalid, Happy – Valid.

**Figure 6:**
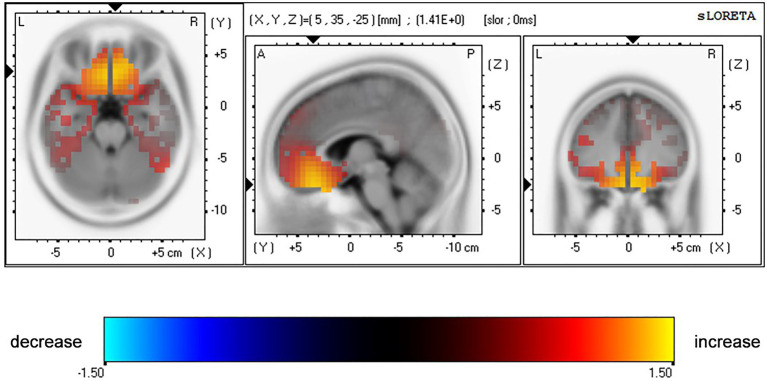
Results of voxel-by-voxel paired *t* tests contrasting current density for the N170 after presentation of a pair of fearful-neutral facial expressions for older versus younger participants. Yellow: older>young subjects. Statistical maps are presented on the MNI space and thresholded at *p*<0.05.

The ANOVA for N170 peak amplitude (Table 4 and Figure 5) also showed a significant main effect of emotion types [*F*(1,44)=6.62, *p*=0.014, *η*^2^=0.13] and a marginally significant four-way interaction among emotion type, cue validity, electrode location, and age [*F*(1,44)=3.93, *p*=0.054, *η*^2^=0.08]. For the younger group, the N170 amplitude following fear-neutral face pairs was larger compared with that following the happy-neutral ones over the left hemisphere [*t*(22)=2.32, *p*=0.030, Cohen’s *d*=0.48]; the older group’s right hemisphere showed similar patterns, although the results were not statistically significant [*t*(22)=1.97, *p*=0.061, Cohen’s *d*=0.41]. For the younger group, a separate analysis of the fear trials showed that the N170 peak amplitude evoked by happy faces was greater over the right hemisphere than over the left hemisphere either under the valid or invalid condition [*t*(22)=2.07, *p*=0.051, Cohen’s *d*=0.43; *t*(22)=2.40, *p*=0.025, Cohen’s *d*=0.50], whereas the older group did not show any hemisphere asymmetry (*Ps*>0.05). Correspondingly, sLORETA independent *t* tests revealed that invalid fearful trials were associated with greater activity in the bilateral ACC/medial frontal gyrus (BA 24, 25, 32), superior frontal gyrus (10, 11), and inferior prefrontal gyrus (47) among older adults compared with younger adults (Table 5 and Figure 6).

**Table 5:** Summary of significant results provided by whole-brain sLORETA analyses for 130–210ms (N170 component) after invalidly cued fearful probes with older versus younger participants.

Close regions (*d*<5mm)	MNI coordinates (mm)			Brodmann area (BA)	Voxels	*t*	*p*	Cohen’s *d*
	x	y	z					
ACC/medial frontal gyrus	5	29	−6	24, 25, 32	36	1.38	0.004	0.41
Superior frontal gyrus	10	38	−10	10, 11	119	1.41	0.005	0.42
Inferior prefrontal gyrus	10	28	−27	47	36	1.41	0.005	0.42

*The close anatomical regions (<5mm) to the maximum, MNI coordinates, and corresponding BAs of extreme values of t are listed. Positive values of t indicate stronger current density for invalidly cued fearful probes among older than younger participants. The numbers of voxels exceeding a threshold of p<0.05 are also reported*.

## Discussion

This study examined whether response inhibition would restrict the availability of cognitive control resources by suppressing prepotent behaviors and then influence the PE seen in go/no-go detection tasks. We opted for a go/no-go detection task, in which participants had to judge but withhold their responses on the no-go trials. Additionally, only the VEPs generated in the no-go trials, which reflect conflict monitoring, were analyzed. Our results showed that when there was no attentional demand, age-related positivity effects were not observed. We believe that the existence of age-related positive effects in a specific paradigm depends on whether the paradigm involves the core element of cognitive control. The study results demonstrated that healthy older adults initially showed attentional bias toward threat-related faces when they withheld a response and that the VEP reliably captured this attention shift at the early attentional stage. In a similar study, age-related PE was reduced in an emotion-induced blindness task when older adults had a working memory overload (Kennedy et al., 2019). These results suggest that PE is a fast-acting bias that occurs within hundreds of milliseconds of the stimulus onset and is mediated by the same cognitive/attentional resources required for response inhibition, which supports the SGSST. In addition, activation of bilateral hemispheres may be important potential signals of normal aging. We will first discuss the behavioral data and then the ERP findings.

### Behavioral Findings

This study used ERPs and behavioral measures to examine the cognitive control mechanisms, especially response inhibition, underlying age-related PE in the attention processing of emotional faces with a go/no-go detection task among healthy younger and older adults. Aligning with previous research (Isaacowitz et al., 2006), we found that the mean RT on go trials of older adults was slower than that of younger adults, suggesting a decline in cognitive performance with age.

Based on signal detection theory, we used the discrimination index *d*' as the measure of accuracy in the go/no-go detection task, with a higher value of *d*' indicating that a signal is more apparent and more easily observed. The *d*' analyses showed that older adults had better discrimination of the bar probe following fearful face pairs than younger adults. An opposite pattern was discovered for happy pairs, such that the value of *d*' of younger adults was higher than that of older adults. Research has suggested that when cognitive control resources are exhausted by additional cognitive load, threat cues irrelevant to the task are more likely to capture attention. The explanation for this phenomenon is that when cognitive control resources are depleted, an individual would face difficulty in suppressing task-irrelevant threat cues processing and find it easier to pay attention. Conversely, if a concurrent cognitive load does not affect cognitive control resources, an individual can block the entry of threat information irrelevant to the task (Holmes et al., 2014). The availability of cognitive resources in older adults is typically less than that in younger adults (Labouvie-Vief, 2009). The study’s go/no-go detection paradigm involved a secondary task that required a response, namely, response inhibition, accompanied by cognitive demand. With cognitive aging, this task may consume all cognitive control resources of older adults but only few among younger adults. Thus, the depletion of available cognitive control resources may result in positive preferences as attention processing fades in older adults, which would be consistent with the idea that a certain amount of top–down/cognitive control ability is necessary for age-related PE.

The participants showed a high sensitivity (values of *d*') to the probe target following the fearful-neutral versus happy-neutral pairs. Threatening faces may capture attention in an involuntary manner (Pourtois et al., 2004). Given that threatening faces can convey important environment-related information, they capture attention relatively easily compared with happy faces, reflecting a threat-detection advantage. We also found that discriminability was enhanced by valid trials relative to the invalid ones. Thus, when the bar replaced a fearful or happy face, the ability of the participants to judge the bar direction increased compared with the case of neutral faces in both visual fields. A viable explanation is that emotional cues are highly biologically relevant, which allows them to be further processed in preparation for the execution of behavioral responses (Brosch et al., 2008). Nevertheless, we found the spatial validity effect (Santesso et al., 2008) to be significantly greater for happy faces than fearful faces. This finding differs from that of earlier research that found greater spatial facilitation in fearful faces among healthy college-aged individuals (Pourtois et al., 2004). Both results are plausible under the assumption that attention is directed toward a relatively more threatening stimulus (Mogg and Bradley, 1999; Cooper et al., 2015).

### ERP Findings

Complementing these behavioral findings were the earliest electrophysiological index of spatial attention manifested in the P1 and N170 (Crognale, 2002; Pourtois et al., 2004; Santesso et al., 2008). Although the P1 and N170 components reflect the neural mechanism for detecting early face processing, different aspects of faces are processed differently. The P1 is assumed to indicate overall face processing, whereas the N170 indicates early facial structure coding (Macchi Cassia et al., 2006). Our results demonstrated that for older and younger adults, the latencies of the P1 and N170 located at the left electrode sites were shorter than those at the right electrode sites in face processing, indicating the superiority of the left hemisphere in face processing speed. In addition, for younger adults, the amplitude of the N170 at the right electrode position was greater than that at the left, whereas older adults showed no hemispheric difference. In other words, younger adults showed right-lateralized face processing intensity (Bentin et al., 1996), whereas older adults showed no lateralization effect. A similar phenomenon has been observed in previous studies. Hemispheric dominance is reported to include speed and intensity dimensions (Peng et al., 2002). The left hemisphere dominates the N170 latency, whereas the right hemisphere dominates the amplitude (Peng et al., 2002). The Hemispheric Asymmetry Reduction in Older Adults Model (HAROLD) suggests that during cognitive tasks, the brain activity of younger adults presents obvious asymmetry, namely, a unilateral advantage, whereas that of older adults shows less asymmetry, manifesting bilateral activation patterns (Cabeza, 2002). Consistent with this model, older adults tend to show bilateral brain activity in the go/no-go task (Simmonds et al., 2008). Lateralization attenuation in brain activation may be another important age-related signal. Indeed, ERP studies have suggested that age-related hemispheric asymmetry decrement might be a functional compensation for the neurocognitive decline associated with aging (Collins and Mohr, 2013).

We found an enhanced N170 amplitude among older adults in response to fearful faces at the left electrode positions, which related to validity effects. Meanwhile, the N170 amplitude of younger adults was not modulated by emotional valence under any condition. This suggests that older adults emphasize negative over positive stimuli. In contrast, younger adults do not show any attentional bias, resulting in a reduced PE in early attention (in the first 250ms of image display). A lack of the age-related PE could be accounted for in the go/no-go detection task involving response inhibition; this indicates that response inhibition would overwhelmingly tax most of older adults’ cognitive resources. Therefore, the cognitive control mechanisms underlying age-related PE occur in early attention.

### Source Localization Findings

Coincidentally, we also found that older adults tended to show increased brain activity in the anterior rostral cingulate area (rACC; BA 24, 25, 32) in response to invalidly cued threatening faces during no-go trials. That is, involvement of response inhibition is consistent with the role of this region in detecting response competition to achieving cognitive control in information processing (e.g., attentional control, response competition, and overriding prepotent responses; Bush et al., 2000; Botvinick et al., 2001).

More specifically, recent studies have reported that older adults have greater activity than younger adults in the anterior cingulate cortex (ACC) and ventromedial PFC during the processing of negative emotional faces (Sakaki et al., 2013; Dolcos et al., 2014). Correspondingly, fMRI studies on age-related differences in emotional processing have shown that, given available cognitive resources, older adults show greater rACC activities than younger adults in happy faces trials (Brassen et al., 2011). Moreover, brain activity in the ACC is linked to age-related PE, and the increment in prefrontal activity in older adults seems to be an indicator of compensatory improvement in cognitive control ability or impairment mechanism in intracortical inhibition (Amato et al., 2016).

It remains unclear when older adults show increased or decreased PFC activation to mediate cognitive control. We observed enhanced ACC activity patterns among older participants during the N170 window, which is a possible answer to this question. Older adults were more inclined to gatekeep negative emotions when their available cognitive resources were reduced, and the recruitment of cognitive control resources occurred in early visual processing. Cognitive neuroscientists over the past decade have documented compensatory cognitive scaffolding when frontal functional engagement increases (Aktürk et al., 2020), accompanied by white matter integrity, cortical thickness, and dopaminergic activities. Moreover, even the functional engagement of the posterior regions, including the occipital area and hippocampus, decreases with aging (Goh and Park, 2009). Aktürk et al. (2020) emphasized that older adults have higher theta phase locking values than their younger counterparts in the frontal regions. This posterior-anterior shift in the aging activation pattern is considered an important cognitive compensatory mechanism with aging (Davis et al., 2008).

Our sample yielded no behavioral evidence of valence differences when using traditional RT measures. One possible explanation is that RT reflects the total cognitive processes from stimulus onset to behavioral response execution (Kappenman et al., 2014). Until the participants made their behavioral response, their attention may have shifted away from the emotional stimulus by the time the target was presented. In contrast to RT, the VEP was more reliable in onset latency and amplitude measures. The VEP indicated that older adults mobilized cognitive control for coping with the go/no-go detection task associated with response inhibition processes *via* engagement of the rACC and PFC during early visual processing. Positive tendencies would not emerge when available cognitive control resources were depleted.

### Limitations and Future Research

The following are the study’s limitations. First, we only focused on healthy older adults. Additionally, although there are contradictory findings on how age-related PE changes along with cognitive decline (Gorenc-Mahmutaj et al., 2015; Leblond et al., 2016), it remains an open question whether the early stage of attention is affected among older adults with cognitive issues. Future studies could use a relatively larger sample that includes individuals with different levels of cognitive abilities to expand on the current findings. Second, we have demonstrated that response inhibition involved in cognitive control resources plays a mediating role in the emergence of PE at an early attentional stage. Future neuroimaging studies may explore more specific neural networks that contribute to this earlier attention processing influence. Third, we used emotional faces as stimuli to examine age differences during attention processing; future studies should emphasize the importance of social backgrounds, such as paradigms outside the laboratory. The ecological validity of laboratory studies would be improved, and ambiguous results would also be reduced. Fourth, the number of trials in our study was lower than the task of Pourtois et al. (2004). This may be one possible explanation for why the results cannot be fully replicated. Finally, it is possible that stimulus features that differ between the images of the faces, rather that emotions, have affected the pre-attentive processing of the faces (Horstmann, 2007). Future studies could use the schematic faces as experimental materials to replicate this study to address this issue.

These findings have several implications for future research based on the CCH of the SGSST. First, in addition to the mechanism-level understanding of attentional prioritization changes with aging, the way cognitive control affects the attentional processing of older adults has implications for how health professionals can help improve cognitive function. When cognitive resources are inaccessible, older adults no longer prioritize positive stimuli, going to the extent of prioritizing negative information over positive information in the early stage of visual attention processing. This tendency indicates the requirement of boosting the amount of accessible cognitive resources through cognitive training in this crucial time window to reverse the negative attention pattern. Second, older adults can engage in daily leisure and entertainment activities (i.e., chess and other puzzle games; Chesham et al., 2017) that can be advocated for them. Tools, such as ERP, enable the consideration of when cognitive training strategies would be restrained in terms of effectiveness. Finally, we applied sLORETA source localization to access cortex activations, including the ACC and medial, superior, and inferior prefrontal gyrus, by measuring the scalp. These cortex activations are presumed to be functional outputs associated with amygdala activation (Blair et al., 1999; Vuilleumier and Pourtois, 2007).

## Data Availability Statement

The original contributions presented in the study are included in the article/supplementary material, and further inquiries can be directed to the corresponding author.

## Ethics Statement

The study was approved by the Institutional Review Board of the Institute of Psychology of the Chinese Academy of Sciences. The patients/participants provided their written informed consent to participate in this study. Written informed consent was obtained from the individual(s) for the publication of any potentially identifiable images or data included in this article.

## Author Contributions

HainL contributed to conception, design of the work, collection of the data, and drafting work. YL, HaihL, and XD contributed to data analysis and interpretation of data. BH revised the work. All authors contributed to the article and approved the submitted version.

## Funding

This work was funded by the Social Science Foundation of Hebei Province (HB21SH020).

## Conflict of Interest

The authors declare that the research was conducted in the absence of any commercial or financial relationships that could be construed as a potential conflict of interest.

## Publisher’s Note

All claims expressed in this article are solely those of the authors and do not necessarily represent those of their affiliated organizations, or those of the publisher, the editors and the reviewers. Any product that may be evaluated in this article, or claim that may be made by its manufacturer, is not guaranteed or endorsed by the publisher.
